# Differences in Postnatal Growth of Preterm Infants in Northern China Compared to the INTERGROWTH-21st Preterm Postnatal Growth Standards: A Retrospective Cohort Study

**DOI:** 10.3389/fped.2022.871453

**Published:** 2022-06-13

**Authors:** Li Zhang, Nan-Nan Gao, Hui-Juan Liu, Qiong Wu, Ju Liu, Ting Zhang, Jin Sun, Jian-Hong Qi, Xiu-Yun Qiao, Yan Zhao, Yan Li

**Affiliations:** ^1^Department of Developmental Pediatrics and Child Health Care, The First Affiliated Hospital of Shandong First Medical University and Shandong Provincial Qianfoshan Hospital, Shandong Engineering and Technology Research Center for Pediatric Drug Development, Jinan, China; ^2^Medical Research Center, Institute of Microvascular Medicine, Shandong Provincial Qianfoshan Hospital, Shandong University, Jinan, China; ^3^School of Clinical Medicine, Weifang Medical University, Weifang, China; ^4^Department of Neonatology, Shandong Provincial Hospital Affiliated to Shandong First Medical University, Jinan, China

**Keywords:** growth reference, growth standard, INTERGROWTH-21st, postnatal growth, preterm infants

## Abstract

**Background:**

The INTERGROWTH-21st preterm postnatal growth standards (IPPGS) have increasingly been used to evaluate the growth of preterm infants worldwide. However, the validity of IPPGS's application to specific preterm populations remains controversial. This retrospective cohort study aimed to formulate reference growth charts for a preterm cohort in northern China and compare them to the IPPGS.

**Methods:**

A total of 1,827 healthy preterm infants with follow-up visits before 70 weeks of postmenstrual age (PMA) were retrospectively sampled from a preterm cohort (*N* = 2,011) born between 1 January 2011 and 28 February 2021, at the First Affiliated Hospital of Shandong First Medical University. Using the Generalized Additive Models for Location, Scale, and Shape method, 5,539 sets of longitudinal data were used to construct percentile and *Z*-score charts of length, weight, and head circumference (HC) at 40–64 weeks of PMA. *Z*-scores of length, weight, and HC (LAZ, WAZ, and HCZ) before 64 weeks were calculated using the IPPGS. Differences in the 50th percentile values between preterm infants and IPPGS (dLength, dWeight, and dHC) were calculated. *Z*-scores were assigned to six PMA clusters: 40–44, 44–48, 48–52, 52–56, 56–60, and 60–64 weeks for comparison between sexes.

**Results:**

For eligible infants, the mean PMA and weight at birth were 33.93 weeks and 2.3 kg, respectively. Boys, late preterm infants, twins, and infants with exclusively breastfeeding accounted for 55.8, 70.6, 27.8, and 45.9%, respectively. Compared to IPPGS, preterm infants were longer and heavier, especially for dLength in girls (range, 2.19–2.97 cm), which almost spanned the 50th and 90th percentiles of IPPGS. The dHC tended to narrow with PMA for both sexes. The mean LAZ, WAZ, and HCZ of both sexes at all PMA clusters were >0, especially for LAZ and WAZ (about 1.0 relative to IPPGS), indicating higher levels than the IPPGS at 40–64 weeks. Girls had larger LAZ at each PMA cluster, larger WAZ at 40–44 weeks, and lower HCZ after 56 weeks than boys. HCZ declined with PMA for both sexes.

**Conclusion:**

Postnatal growth of this preterm cohort was considerably higher than that of the IPPGS at 40–64 weeks of PMA with sex differences.

## Introduction

Prematurity is the leading cause of neonatal mortality worldwide (1). It contributes to 26% of neonatal causes of death in China (2). Despite the increased survival rate with advances in prenatal and neonatal care (3, 4), these surviving preterm infants are at an increased risk of hypertension, metabolic syndrome, and impaired neurodevelopment (5–7). Consequently, a full understanding of optimal postnatal growth is a prerequisite for tailoring adequate supportive treatments to improve short-term and long-term outcomes in preterm infants (8). This requires robust growth charts to monitor whether preterm infants have potentially abnormal growth that might be indicative of adverse health conditions (9). However, the selection of growth charts has always been controversial, given the lack of consensus regarding the most suitable charts to use (10, 11).

Currently, the most widely implemented clinical practice in assessing the postnatal growth of preterm infants is the plotting of anthropometric measurements on Fenton charts before 50 weeks of postmenstrual age (PMA) and plotting on WHO standard charts after 40–50 weeks (12). However, this strategy has several known limitations. First, Fenton charts are not the actual postnatal growth trajectories of preterm infants since they are artificially smoothened growth curves from cross-sectional birth data on preterm infants and WHO data on term infants at 50 weeks of PMA (13, 14). Second, WHO standards were established based on data on term infants and might not be the suitable growth targets for preterm infants (15). Furthermore, this strategy artificially divides the continuous postnatal growth of preterm infants into two stages i.e., before and after corrected term age and specific evaluation with two different charts.

In 2015, the INTERGROWTH-21st project claimed that they developed the prescribed international preterm postnatal growth standards (IPPGS) before 64 weeks of PMA from longitudinal growth data of 201 singleton preterm infants (16). Recently, IPPGS has increasingly been used in the clinical practice of growth assessment for preterm infants in various countries (10, 17–21). However, little attention has been paid to the investigation of the difference between the actual postnatal growth in a specific preterm population and the IPPGS. In addition, data on IPPGS use in Chinese preterm infants is scarce (20, 21). Nevertheless, whether the actual postnatal growth of a specific preterm population differs from that of the IPPGS determines the interpretation of the growth assessment results with the IPPGS and subsequent clinical decisions, such as the optimal time point to terminate fortified nutrition. In addition, our previous studies demonstrated higher growth levels among preterm infants in Shandong, China, than those indicated by the Fenton reference and WHO standards before the corrected age of 2 years old (22, 23). Therefore, we hypothesized that this preterm population might also have higher postnatal growth levels than those of the IPPGS.

This study aimed to establish growth charts for length, weight, and head circumference (HC) at 40–64 weeks of PMA using longitudinal data of a preterm cohort in northern China. Furthermore, the study sought to compare growth charts of this preterm cohort with those of the IPPGS to elucidate the difference between the postnatal growth of preterm infants in northern China and the new international preterm growth standards and to provide information on a reasonable interpretation of growth assessment, nutrition, and health status using the IPPGS.

## Materials and Methods

The study retrospectively collected the longitudinal anthropometric data (i.e., length, weight, and HC) of eligible preterm infants from the database of a cohort of preterm neonates born between 1 January 2011 and 28 February 2021, and had follow-up visits at the First Affiliated Hospital of Shandong First Medical University in Jinan, China, before the deadline of data analysis (7 August 2021). The study protocol was reviewed and approved by the Medical Ethics Committee of the First Affiliated Hospital of Shandong First Medical University. Parents of all participants provided written informed consent.

**Figure 1:**
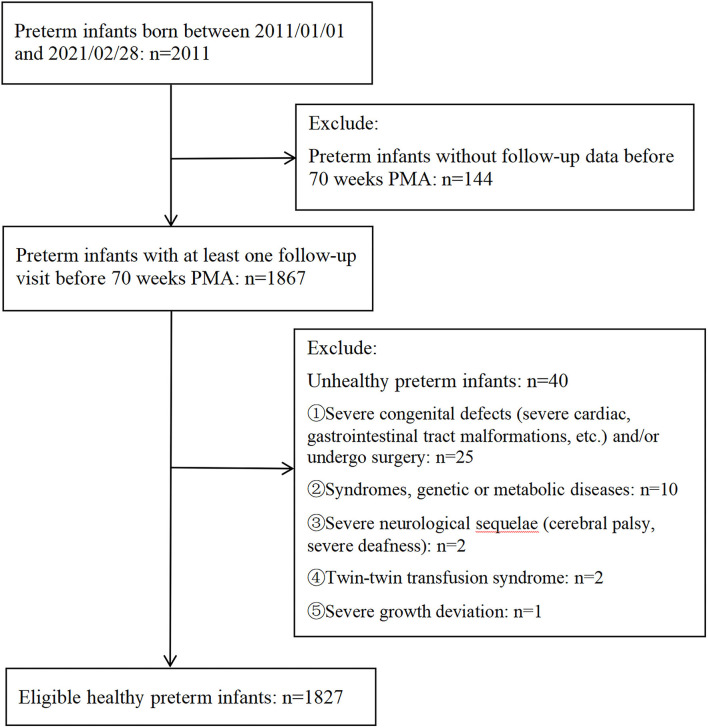
Flowcharts of sampling eligible healthy preterm infants. Severe growth deviation in this study was identified as the difference between the *Z*-scores of two adjacent measurements >+2 or <-2 according to the Fenton reference (before 40 weeks of PMA) and WHO growth standards (40 weeks of PMA and beyond). PMA, postmenstrual age.

The eligibility criteria were as follows: (1) Preterm birth: PMA at birth ≤36 weeks; (2) ≥1 follow-up visits before 70 weeks of PMA; and (3) No major congenital malformations, syndromes, surgeries, major central nervous system sequelae, twin–twin transfusion syndrome, and severe growth deviation. Severe growth deviation in this study was defined as the difference between the *Z*-scores of two adjacent measurements > +2 or <-2 according to the Fenton reference (before 40 weeks of PMA) and WHO growth standards (≥40 weeks of PMA). A flowchart of the sampling process for eligible healthy preterm infants is shown in Figure 1.

The feeding practices during the early postnatal period for preterm infants were based on the Nutrition Practice Care Guidelines for Preterm Infants in the Community (2013) and the Chinese Society of Parenteral and Enteral Nutrition (CSPEN) Guidelines for Nutrition Support in Neonates (24, 25). Before discharge, the preterm infants were fed according to their nutrition risks, as described in detail in our previous study (22). After discharge (when infants weighed ≥2,000 g target weight with stable feeding and body temperature), the parents were encouraged to breastfeed their babies without fortification (standard infant formula was used in cases of insufficient breast milk).

The parental baseline data collected were as follows: age, ethnicity, maternal obstetric history, and mode of delivery. The data on the infants were as follows: sex, PMA at birth (week), length (cm), and weight (kg) at birth (HC was not measured at birth), singleton or multiple births, and feeding modes at the first visit. These data were collected from questionnaires completed by parents at the first follow-up visit.

Follow-up visits were scheduled at term (40 weeks of PMA) and at 1, 3, 4, 5, 6, 9, and 12 months of corrected age. Anthropometric measurements were taken at each follow-up visit by two trained staff members. Length and weight were measured using an instrument constituted by an infantometer and an electronic scale (“KANGWA” WS-RTG-1G, Suzhou, China; length range, 30–105 cm, with digit counter readings precise to 1 mm; weight range, 0–60 kg calibrated to 0.05 kg). HC was measured with a tape measure replaced once a month (“WenTai,” Infant HC Tape Measure, Foshan, China; range, 0–56 cm, with digit counter readings precise to 1 mm). Each staff member independently measured and recorded a complete set of measurements. Thereafter, the two staff members compared their readings and recorded the mean of each pair of readings. The maximum acceptable differences were as follows: length, 7 mm; weight, 100 g; and HC, 5 mm.

The *Z*-scores of growth parameters at birth and each follow-up visit were calculated using the INTERGROWTH-21st Newborn Size References/Standards (INSR/S; 24–42 weeks of PMA) application and the IPPGS application (26), respectively. Growth charts of percentiles (the 3rd, 10, 25, 50, 75, 90, and 97th percentiles denoted as 3, 10, 25, 50, 90, and P97) and *Z*-scores (−3,−2,−1, 0, 1, 2, and 3) of length, weight, and HC of the preterm infants at 40–64 weeks of PMA stratified by sex were constructed using Generalized Additive Models for Location, Scale, and Shape (GAMLSS) in R software (version 3.6.1). The selection of the GAMLSS model for each growth parameter was based on the Akaike information criterion (AIC), the Bayesian Information Criterion (BIC), or Schwarz Bayesian Criterion (SBC) (27, 28). The final selected models are shown in Supplementary Table 1.

The differences in P50 values between the preterm infants and IPPGS were calculated by subtracting the P50 values of IPPGS from those of preterm infants by sex. The *Z*-scores were assigned to six PMA clusters: ≥40 & <44, ≥44 & <48, ≥48 & <52, ≥52 & <56, ≥56 & <60, and ≥60 & <64 weeks. The *Z*-scores were compared between boys and girls using independent-sample *t*-tests. Additionally, we observed whether the *Z*-scores were >0 (indicating higher growth levels than those of the IPPGS). Statistical analyses were performed using SPSS software version 21 (IBM Corporation, Armonk, NY, United States). Continuous variables are presented as means (SD), while categorical variables are presented as frequency (*n*) and percentage (%). The level of statistical significance was set at *P* < 0.05.

## Results

### Baseline Characteristics

A total of 1,827 eligible preterm infants were sampled from the preterm cohort (*N* = 2,011). Altogether, 5,539 sets of longitudinal anthropometric measurements (each including length, weight, and HC) of eligible preterm infants before 70 weeks of PMA (not including data at birth) were used to construct the growth charts. The mean follow-up visits were 3.03 visits for each infant. The baseline characteristics are shown in Table 1.

**Table 1:** Baseline characteristics of 1,827 preterm infants^a^.

	**Mean (SD) or *N* (%)**
Sex	
Boy	1,019 (55.8%)
Girl	808 (44.2%)
PMA at birth (weeks)	33.93 (0.06)
Birth Length (cm)	45.11 (0.10)
Birth Weight (kg)	2.30 (0.02)
Birth LAZ (INSR/S)^§^	0.37 (0.03)
Birth WAZ (INSR/S)^§^	0.23 (0.02)
N. of Gestation	
1^st^ Gestation	886 (48.5%)
2^nd^ Gestation	590 (32.3%)
≥3^rd^ Gestation	351 (19.2%)
N. of parity	
Primipara	936 (51.2%)
2^nd^ Parity	786 (43.0%)
≥3^rd^ Parity	105 (5.8%)
N. of fetus	
Singleton	1,316 (72.0%)
Twin	508 (27.8%)
Triplet	3 (0.2%)
Delivery mode	
Cesarean section	1,255 (68.7%)
Vaginal	572 (31.3%)
Maternal age (year)	31.90 (0.11)
Paternal age (year)	33.40 (0.12)
Ethnicity	
Han	1,818 (99.5%)
Other	9 (0.5%)
Subgroups by PMA at birth	
Extremely preterm (≤ 28 weeks)	113 (6.2%)
Moderate preterm (29–33 weeks)	424 (23.2%)
Late preterm (34–36 weeks)	1,290 (70.6%)
Intrauterine growth status by birth weight percentiles (INSR/S)^§^	
SGA (< P10)	117 (6.4%)
AGA (P10–90)	1,488 (81.4%)
LGA (>P90)	221 (12.1%)
Feeding mode at first visit	
Breast feeding	838 (45.9%)
Mixed feeding	832 (45.5%)
Artificial feeding	157 (8.6%)

a *AGA, appropriate for gestational age; HCZ, Z-score of head circumference; INSR/S, INTERGROWTH-21st International Newborn Size Standards/Reference; LAZ, Z-score of length; LGA, large for gestational age; PMA, postmenstrual age; WAZ, Z-score of weight; SGA, small for gestational age*.

§ *Z-scores of a boy born at 23 weeks of PMA could not be calculated according to the INSR/S (range of PMA in INSR/S: 24–42 weeks)*.

The mean PMA at birth was 33.93 (0.06) weeks (range, 23–36 weeks). In the cohort, 70.6% of infants were late preterm and 72% were singletons. Most infants had a Han ethnicity (99.5%) and were breastfeeding at their first follow-up visit (91.1%). The LAZ and WAZ at birth according to the INSR/S (26) were >0. Based on INSR/S birth weight percentiles, the proportions of infants who were small for gestational age (SGA; < P10) and large for gestational age (LGA; >P90) were 6.1% and 12.1%, respectively.

### Growth Charts for Preterm Infants

Growth charts of percentiles (P3, P10, P25, P50, P75, P90, and P97) of length, weight, and HC for the preterm infants stratified by sex at 40–64 weeks of PMA are shown in Supplementary Table 2 and Supplementary Figure 1. Growth charts of *Z*-scores (−3,−2,−1, 0, 1, 2, and 3) are shown in Supplementary Table 3.

### Comparison of Growth Levels Between Preterm Infants and IPPGS

#### Difference Values of P50 Between Preterm Infants and IPPGS

**Figure 2:**
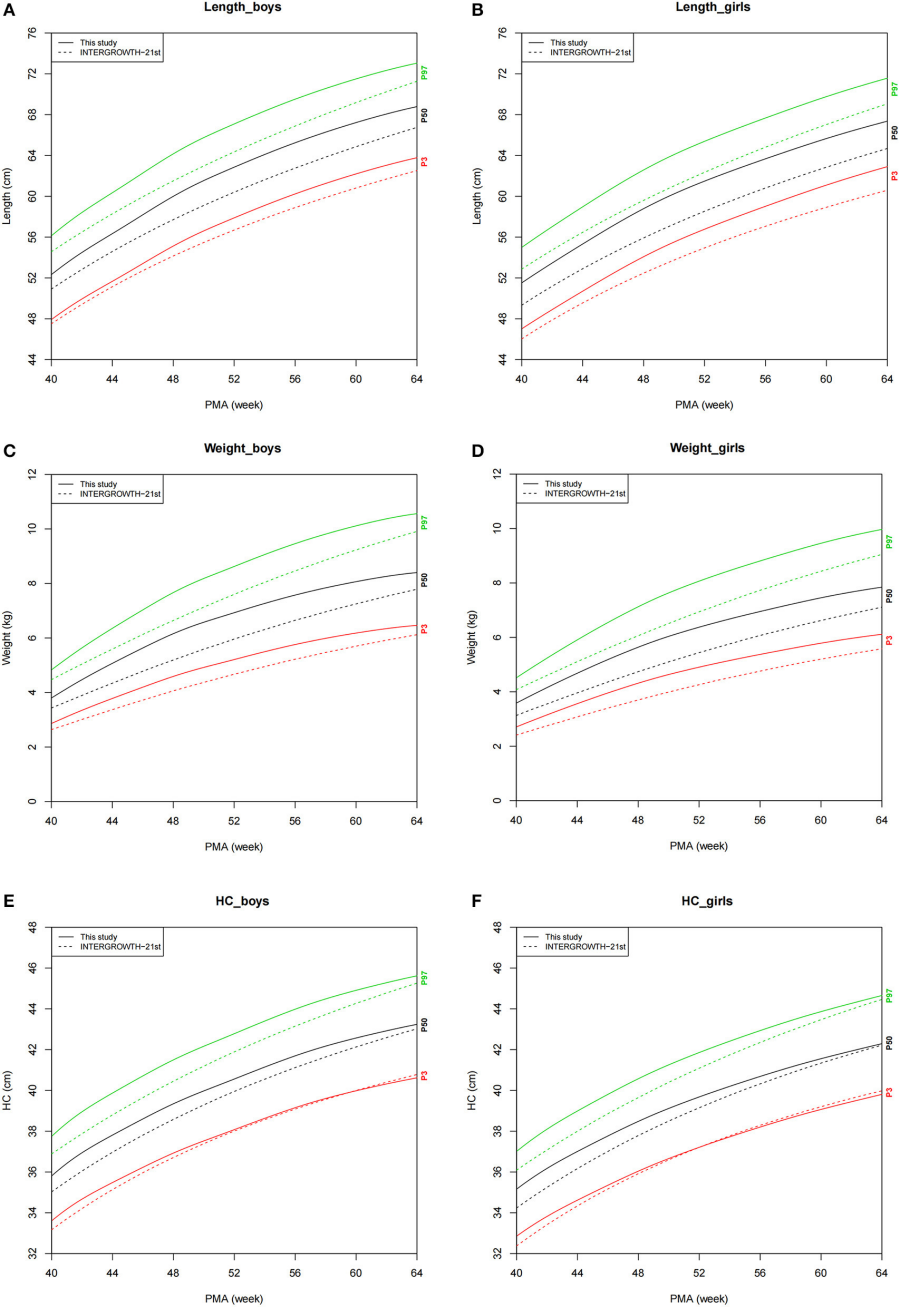
Comparison of growth charts (P3, P50, and P97) between the preterm infants and IPPGS. **(A)** Length_boys; **(B)** Length_girls; **(C)** Weight_boys; **(D)** Weight_girls; **(E)** HC_boys; **(F)** HC_girls. HC, head circumference; IPPGS, INTERGROWTH-21st Preterm Postnatal Growth Standards; PMA, postmenstrual age; P3, P50, and P97, the 3rd, 50th, and 97th percentiles.

The growth charts for P3, P50, and P97 of preterm infants and IPPGS are demonstrated in Figure 2. The P3, P50, and P97 of length and weight and P50 and P97 of HC were all higher at 40–64 weeks of PMA than those of the IPPGS for both sexes. Before and around 50 weeks, the P3 of HC was higher than that of the IPPGS but similar to that of the IPPGS after 50 weeks.

The difference in P50 values of each growth parameter (denoted as dLength, dWeight, and dHC) between preterm infants and IPPGS showed that dLength for girls (range, 2.19–2.97 cm) was consistently larger than that for boys (range, 1.42–2.48 cm). The dWeight for girls (range, 0.46–0.94 kg) and boys (range, 0.37–0.99 kg) were quite consistent. The dHC decreased from 0.94 to 0.07 cm for girls and 0.90 to 0.22 cm for boys with PMA.

#### Z-Scores of the Preterm Infants According to IPPGS

The *Z*-scores of the preterm infants at 40–64 weeks of PMA according to IPPGS are shown in Table 2. The mean *Z*-scores of all parameters were >0. The mean LAZ was 0.82–1.12 for boys and 1.13–1.44 for girls. The mean WAZ was 0.69–1.20 for boys and 0.81–1.27 for girls. The mean HCZ decreased from 0.88 to 0.27 for boys and 0.91 to 0.10 for girls with PMA.

**Table 2:** *Z*-scores of the preterm infants according to the IPPGS^a^.

**PMA cluster (weeks)**	**N**		**PMA (weeks)**		**LAZ**		**WAZ**		**HCZ**	
	**Boy**	**Girl**	**Boy**	**Girl**	**Boy**	**Girl**	**Boy**	**Girl**	**Boy**	**Girl**
≥40 & <44	632	506	41.88 (0.93)	41.83 (0.97)	0.82 (1.15)^¶^	1.13 (1.10)	0.93 (1.01)^*^	1.07 (0.96)	0.88 (1.16)	0.91 (1.12)
≥44 & <48	274	195	46.12 (1.24)	46.14 (1.21)	0.93 (1.24)^§^	1.26 (1.22)	1.15 (1.09)	1.24 (1.00)	0.74 (1.26)	0.63 (1.25)
≥48 & <52	476	367	49.75 (1.08)	49.79 (1.13)	1.12 (1.18)^¶^	1.44 (1.19)	1.20 (1.12)	1.27 (1.06)	0.64 (1.12)	0.60 (1.22)
≥52 & <56	429	326	53.83 (1.04)	53.88 (1.09)	1.11 (1.09)^*^	1.32 (1.11)	1.04 (1.03)	1.13 (1.02)	0.54 (1.20)	0.40 (1.10)
≥56 & <60	399	316	57.91 (1.12)	57.89 (1.01)	1.06 (1.05)^§^	1.27 (1.04)	0.90 (0.99)	0.99 (1.04)	0.48 (1.04)^*^	0.29 (1.13)
≥60 & <64	429	333	61.99 (1.01)	62.12 (1.00)	0.90 (1.06)^¶^	1.19 (1.00)	0.69 (1.01)	0.81 (1.02)	0.27 (1.14)^*^	0.10 (1.06)

a *HCZ, Z-score of head circumference; IPPGS, INTERGROWTH-21st Preterm Postnatal Growth Standards; LAZ, Z-score of length; PMA, postmenstrual age; WAZ, Z-score of weight. Values are presented as mean (SD). Independent-sample t-tests were used to compare values between subgroups of boys and girls. Significant differences between subgroups are marked as*

* *P < 0.05*,

§ *P < 0.01*,

¶ *P < 0.001*.

No difference in the mean PMA was recorded between both sexes in each PMA cluster. Girls had larger LAZ at each PMA cluster than boys (*P* < 0.05). Girls had a slightly larger mean WAZ at each PMA cluster than boys; the statistical difference existed only at ≥40 & <44 weeks of PMA cluster (*P* < 0.05). Girls had similar mean HCZ before 56 weeks as boys had and lower HCZ than boys after 56 weeks (*P* < 0.05).

## Discussion

This study constructed the longitudinal growth charts for length, weight, and HC of a specific preterm cohort from 2011 to 2021 in northern China using the GAMLSS method. It systematically compared these charts with those of the IPPGS stratified by sex and revealed the large disparity between them.

Three methods are commonly used to construct child growth reference curves, namely, cubic splint function, locally weighted regression and smoothing scatterplots, and coefficient of skewness–median–coefficient of variation (LMS) (29). GAMLSS is an emerging method for constructing reference curves for child growth. When modeling variables, such as age and sex, GAMLSS uses all the data in the model. Therefore, the distribution curve tends to be stable, even if the sample size is small. GAMLSS was chosen to create these growth charts because it had a more accurate prediction ability, a smoother curve, and more effective use in our previous study (23, 29) and the other studies in China (30, 31) and other countries (32, 33). Our reference data showed that the data distribution was well-fitted in GAMLSS using the Q-Q plot, worm plot, and residual plot.

Overall, all the growth parameters of the preterm infants—especially length and weight—were considerably larger than those of the IPPGS. The most apparent difference existed in the length of preterm girls; the difference in length between the preterm girls and the IPPGS (range, 2.28–2.82 cm) at 40–64 weeks of PMA almost spanned the 50 and 90th percentile curves of the IPPGS (range, 2.19–2.97 cm). Although the HC of preterm infants was larger than that of the IPPGS at about 40 weeks of PMA, it rapidly approached that of the IPPGS with increasing PMA. The *Z*-scores of all growth parameters for preterm infants, namely, LAZ and WAZ (about 1.0 relative to IPPGS), were >0 at 40–64 weeks of PMA, which further demonstrates the higher growth level of our preterm infants than that of the IPPGS.

The following reasons may account for some of the differences between IPPGS and growth parameters in our study.

First, differences in the design and methodology were present between these two studies: (1) Study design: The IPPGS was constructed using a prescriptive approach to describe normal preterm growth in eight geographically diverse populations, based on longitudinal growth data of 201 healthy singleton preterm infants (16). Our study used longitudinal growth data of a relatively healthy preterm cohort in northern China with much larger sample size; (2) Nutrition and feeding practice: Nutrition and feeding practice are essential for the postnatal growth of preterm infants. Both our study and IPPGS implemented standardized feeding practices and promoted exclusive breastfeeding (16). Due to great heterogeneity in gestational age, birth weight, and neonatal morbidity in a preterm cohort, this study did not document detailed data on the amount and quality of nutrient intake during hospitalization. Instead, we recorded the implementation of feeding practice at the first follow-up visit (around corrected term age), and found that 45.9% of preterm infants were exclusively breastfed, 45.5% were mixed feeding, and 8.6% were complete formula feeding. Since there are no published data on nutrition and feeding practices during hospitalization and after discharge in the study of IPPGS, it is unclear whether the apparent difference between the two studies was due to nutrition and feeding practices; and (3) Twins and triplets: IPPGS only recruited singletons, while twins and triplets were not excluded in this study. Our unpublished data showed that twins and triplets had similar postnatal growth as that of singletons since corrected term age. Furthermore, this study aimed to depict the actual growth trajectory of a preterm cohort, while twins and triplets accounted for a rather high proportion of the preterm population (27.8% in this study). Overall, the IPPGS established the postnatal growth standard of preterm infants, reflecting how they should grow, while this study created a growth reference, reflecting the actual growth level of contemporary preterm infants in Shandong, China. Therefore, the differences in the design and methodology between the two studies may explain some of the disparities in growth.

Second, differences existed in physical growth among different populations. The promotion of the claimed international growth standards, including the WHO growth standards and the newly established IPPGS (16) is based on the assumption that no differences in growth exist internationally or regionally when conditions are optimal (34–36). However, previous studies have proposed that racial or ethnic disparities in human growth result in significant variations in anthropometric parameters among different populations and challenge the use of international standards in specific populations (34, 37–39). In China, the 2005 and 2015 National Growth Surveys demonstrated higher growth levels of Chinese term infants than WHO standards during the first 3 years of life (40, 41). Although the IPPGS was constructed based on prescribed longitudinal data of eight geographically defined populations, the sample size (201 singleton preterm infants) might not be large enough for reliable conclusions as to whether differences existed among different populations (16). In addition, the definition of “healthy” preterm infants is more difficult and controversial than the term infants because preterm infants are prone to neonatal complications. The smaller the gestational age at birth, the more pronounced the effect of neonatal morbidity on postnatal growth. Recently, emerging studies have proposed that disparities exist in growth between specific preterm populations and international growth standards/references. For example, our previous study proved that singleton preterm infants had higher growth levels than that of WHO standards but had similar growth levels to term infants in the same center from corrected term to 2 years of age (23). A multicenter cohort study conducted in Sichuan, China, demonstrated that preterm infants had higher growth levels than those of the IPPGS and WHO standards and had consistent growth with their term counterparts after 3 months of corrected age (21). In addition, our previous study of late preterm infants demonstrated increased growth compared to the widely used Fenton reference during birth and corrected term age (22). These results again raised the ever-existing dispute regarding whether a single growth standard can be representative of child growth regardless of ethnicity, region, or country of origin (34, 37, 38) in the field of postnatal growth of the preterm population.

Third, the influence of early nutrition, especially protein intake, on postnatal growth should not be ignored. Previous studies have elucidated that early-life nutrition, especially protein intake, has crucial effects on postnatal growth in the preterm infants (42, 43). As with the IPPGS, our infants received standardized, evidence-based clinical care and followed current feeding recommendations based on exclusive/predominant breastfeeding (9, 16). However, our study did not document the detailed amount and quality of nutrient intake, especially protein intake, during hospitalization. Therefore, further studies are needed to explore whether the differences in preterm postnatal growth between the two studies are related to the differences in early-life nutrition. Particularly, based on the accepted correlation between protein intake and linear growth (44, 45), whether the length advantage of our preterm girls was correlated to protein intake needs further exploration.

This study had some limitations. First, since this study aimed to investigate the postnatal growth of preterm infants in the real world, only severe perinatal diseases and complications that might significantly affect postnatal growth, such as severe congenital malformations, genetic and metabolic diseases, and necrotizing enterocolitis, which underwent surgery, were documented and excluded. Other neonatal complications, such as neonatal asphyxia, neonatal respiratory distress syndrome, hypoglycemia, and intracranial hemorrhage, were not excluded from this study. Second, the lack of detailed data on the amount and quality of nutrient intake during hospitalization led to the inadequacy to evaluate the effect of early nutrition on postnatal growth. Furthermore, although we recorded the feeding practice at the first follow-up visit, we did not specifically document the amount and energy of formula in preterm infants' mixed feeding and complete formula feeding, which can exert an important influence on the postnatal growth of preterm infants. Third, long-term follow-up evidence of health outcomes for this postnatal growth pattern of preterm infants is needed, considering the potential risk of metabolic diseases later in life related to inappropriate growth in early life (46). Fourth, because 70.6% of the preterm infants were late preterm infants, our study might not be sufficiently powered to evaluate the specific growth of extremely preterm, moderate preterm, and extremely low birth weight/very low birth weight (ELBW/VLBW) infants.

## Conclusion

Our study demonstrated the higher postnatal growth of preterm infants in northern China than that of the new international growth standards for preterm infants, IPPGS. It corroborated the possibility of ethnic and regional disparities in the postnatal growth of preterm infants. Therefore, this study proposes the cautious and rational interpretation of assessment results in the application of IPPGS to a specific preterm population. However, higher growth levels do not necessarily equate to superior growth. The multiple and complicated etiologies for higher postnatal growth among our preterm cohort need further exploration.

## Data Availability Statement

The original contributions presented in the study are included in the article/Supplementary Material, further inquiries can be directed to the corresponding author/s.

## Ethics Statement

The studies involving human participants were reviewed and approved by Medical Ethics Committee of the First Affiliated Hospital of Shandong First Medical University. Written informed consent to participate in this study was provided by the participants' legal guardian/next of kin.

## Author Contributions

LZ made major contributions to the conception and design of the study, acquisition, analysis, interpretation of the data, and writing and revision of the manuscript. N-NG, H-JL, QW, TZ, JS, X-YQ, and YZ contributed to the acquisition and analysis of data. JL made important contributions to the revision of the manuscript. J-HQ contributed to the analysis of data. YL made substantial contributions to the supervision and project administration of the study and the writing and revision of the manuscript. All authors read and approved the final manuscript.

## Funding

This work was supported by Natural Science Foundation of Shandong Province (Grant No. ZR2021MH296). The funding sources were not involved in the design of the study and collection, analysis, or interpretation of data or in writing the manuscript.

## Conflict of Interest

The authors declare that the research was conducted in the absence of any commercial or financial relationships that could be construed as a potential conflict of interest.

## Publisher's Note

All claims expressed in this article are solely those of the authors and do not necessarily represent those of their affiliated organizations, or those of the publisher, the editors and the reviewers. Any product that may be evaluated in this article, or claim that may be made by its manufacturer, is not guaranteed or endorsed by the publisher.
