# Supplementary material for: Differences in Postnatal Growth of Preterm Infants in Northern China Compared to the INTERGROWTH-21st Preterm Postnatal Growth Standards: A Retrospective Cohort Study
Source: Front Pediatr. 2022 Jun 13;10:871453. doi: 10.3389/fped.2022.871453 (PMC9234397; doi:10.3389/fped.2022.871453)
Supplement: Supplementary file 1 [file Table_1.DOCX]

Supplementary Table 1 GAMLSS models of preterm infants stratified by sex^△*^.

| Growth Parameters | Boys | Girls |
| --- | --- | --- |
| Length | BCPEo | BCCGo |
| Weight | BCCGo | BCCGo |
| HC | BCTo | BCPEo |

△Abbreviation: BCCGo, Box-Cox Cole-Green orig. ; BCPEo: Box-Cox power exponential distribution orig. ; BCTo: Box-Cox t orig. ; GAMLSS: Generalized Additive Models for Location, Scale and Shape; HC: Head circumference.

* Model selection was according to the Akaike information criterion (AIC) and the Bayesian information criterion (BIC) or Schwarz Bayesian criterion (SBC).
