# Supplementary material for: Differences in Postnatal Growth of Preterm Infants in Northern China Compared to the INTERGROWTH-21st Preterm Postnatal Growth Standards: A Retrospective Cohort Study
Source: Front Pediatr. 2022 Jun 13;10:871453. doi: 10.3389/fped.2022.871453 (PMC9234397; doi:10.3389/fped.2022.871453)
Supplement: Supplementary file 14 [file Table_14.DOCX]

Supplementary Material

# Supplementary Figures

**Supplementary Figure 1. Growth charts of percentiles of Length, Weight, and HC for the preterm infants stratified by sex at 40–64 weeks PMA^∆^ .**

1. Length_boys; (B) Length_girls; (C) Weight_boys; (D) Weight_girls; (E) HC_boys; (F) HC_girls

∆Abbreviations: HC, Head Circumference; PMA, postmenstrual age; P3, P10, P25, P50, P75, P90, and P97, the 3rd, 10th, 25th, 50th, 75th, 90th, and 97th percentiles.

# Supplementary Tables

## Supplementary Table 1. GAMLSS models of preterm infants stratified by sex^∆*^.

∆Abbreviation: BCCGo, Box-Cox Cole-Green orig. ; BCPEo: Box-Cox power exponential distribution orig. ; BCTo: Box-Cox t orig. ; GAMLSS: Generalized Additive Models for Location, Scale and Shape; HC: Head circumference.

*Model selection was according to the Akaike information criterion (AIC) and the Bayesian information criterion (BIC) or Schwarz Bayesian criterion (SBC).

## Supplementary Table 2. Growth charts of percentiles of Length, Weight, and HC for the preterm infants stratified by sex at 40–64 weeks PMA**^∆^** .

1. Length_boys; (B) Length_girls; (C) Weight_boys; (D) Weight_girls; (E) HC_boys; (F) HC_girls

∆Abbreviations: HC, Head Circumference; PMA, postmenstrual age; P3, P10, P25, P50, P75, P90, and P97, the 3rd, 10th, 25th, 50th, 75th, 90th, and 97th percentiles.

## Supplementary Table 3. Growth charts of Z-scores of Length, Weight, and HC for the preterm infants stratified by sex at 40–64 weeks PMA**^∆^** .

1. Length_boys; (B) Length_girls; (C) Weight_boys; (D) Weight_girls; (E) HC_boys; (F) HC_girls

∆Abbreviations: HC, Head Circumference; PMA, postmenstrual age.
