# Supplementary material for: Differences in Postnatal Growth of Preterm Infants in Northern China Compared to the INTERGROWTH-21st Preterm Postnatal Growth Standards: A Retrospective Cohort Study
Source: Front Pediatr. 2022 Jun 13;10:871453. doi: 10.3389/fped.2022.871453 (PMC9234397; doi:10.3389/fped.2022.871453)
Supplement: Supplementary file 15 [file Data_Sheet_1.pdf]

# *Supplementary Material*

## 1 Supplementary Figures

**Supplementary Figure 1. Growth charts of percentiles of Length, Weight, and HC for the preterm infants stratified by sex at 40‑64 weeks PMA$^{\Delta}$.**

(A) Length_boys; (B) Length_girls; (C) Weight_boys; (D) Weight_girls; (E) HC_boys; (F) HC_girls

ΔAbbreviations: HC, Head Circumference; PMA, postmenstrual age; P3, P10, P25, P50, P75, P90, and P97, the 3rd, 10th, 25th, 50th, 75th, 90th, and 97th percentiles.

## 2 Supplementary Tables

### 2.1 Supplementary Table 1. GAMLSS models of preterm infants stratified by sex$^{\Delta *}$.

ΔAbbreviation: BCCGo, Box-Cox Cole-Green orig. ; BCPEo: Box-Cox power exponential distribution orig. ; BCTo: Box-Cox t orig. ; GAMLSS: Generalized Additive Models for Location, Scale and Shape; HC: Head circumference.

*Model selection was according to the Akaike information criterion (AIC) and the Bayesian information criterion (BIC) or Schwarz Bayesian criterion (SBC).

### 2.2 Supplementary Table 2. Growth charts of percentiles of Length, Weight, and HC for the preterm infants stratified by sex at 40–64 weeks PMA$^{\Delta}$.

(A) Length_boys; (B) Length_girls; (C) Weight_boys; (D) Weight_girls; (E) HC_boys; (F) HC_girls

ΔAbbreviations: HC, Head Circumference; PMA, postmenstrual age; P3, P10, P25, P50, P75, P90, and P97, the 3rd, 10th, 25th, 50th, 75th, 90th, and 97th percentiles.

### 2.3 Supplementary Table 3. Growth charts of Z-scores of Length, Weight, and HC for the preterm infants stratified by sex at 40–64 weeks PMA$^{\Delta}$.

(A) Length_boys; (B) Length_girls; (C) Weight_boys; (D) Weight_girls; (E) HC_boys; (F) HC_girls

ΔAbbreviations: HC, Head Circumference; PMA, postmenstrual age.
