# Supplementary figures and images for: Differences in Postnatal Growth of Preterm Infants in Northern China Compared to the INTERGROWTH-21st Preterm Postnatal Growth Standards: A Retrospective Cohort Study
Source: Front Pediatr. 2022 Jun 13;10:871453. doi: 10.3389/fped.2022.871453 (PMC9234397; doi:10.3389/fped.2022.871453)

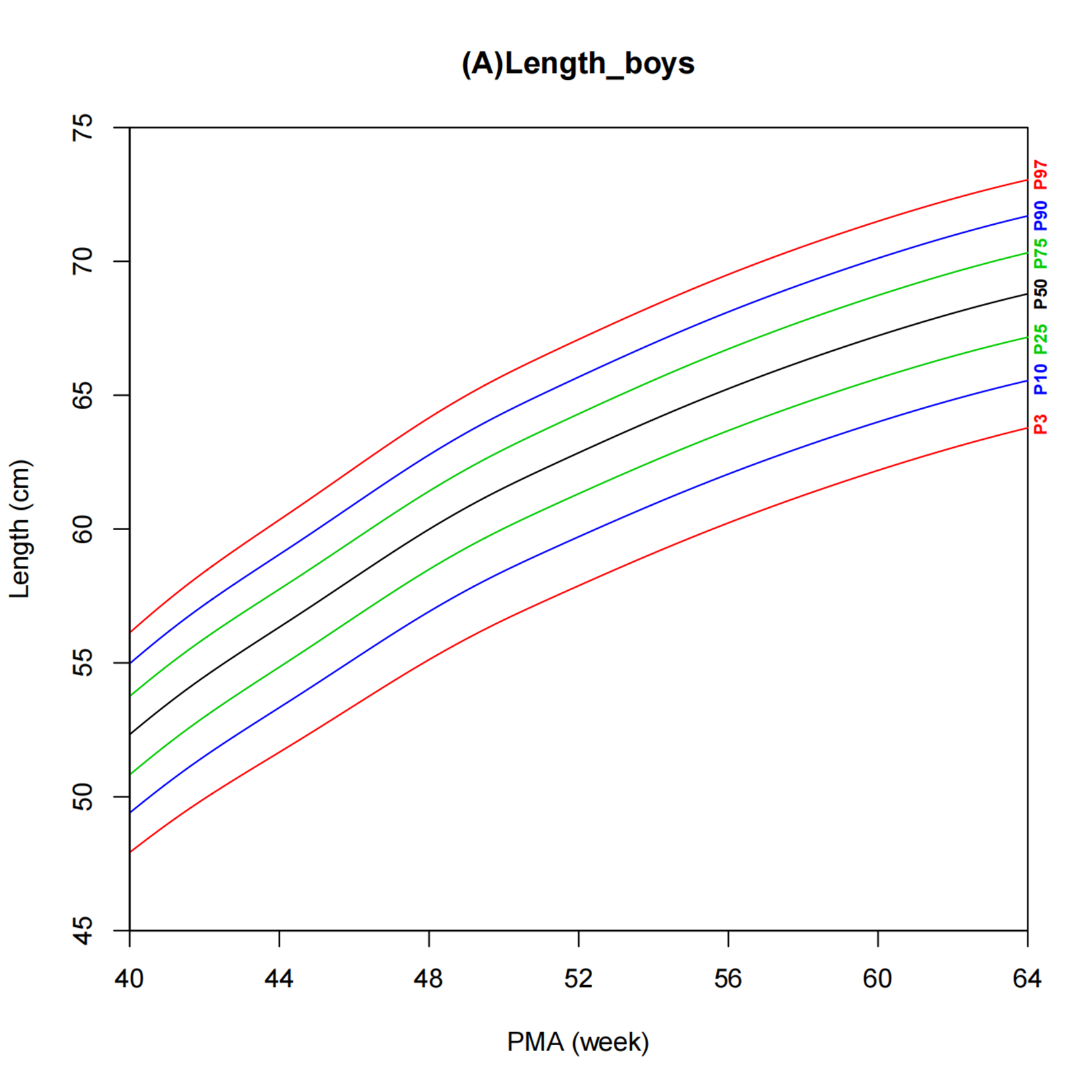

Supplement: Supplementary file 16 [file Image_1.TIF]

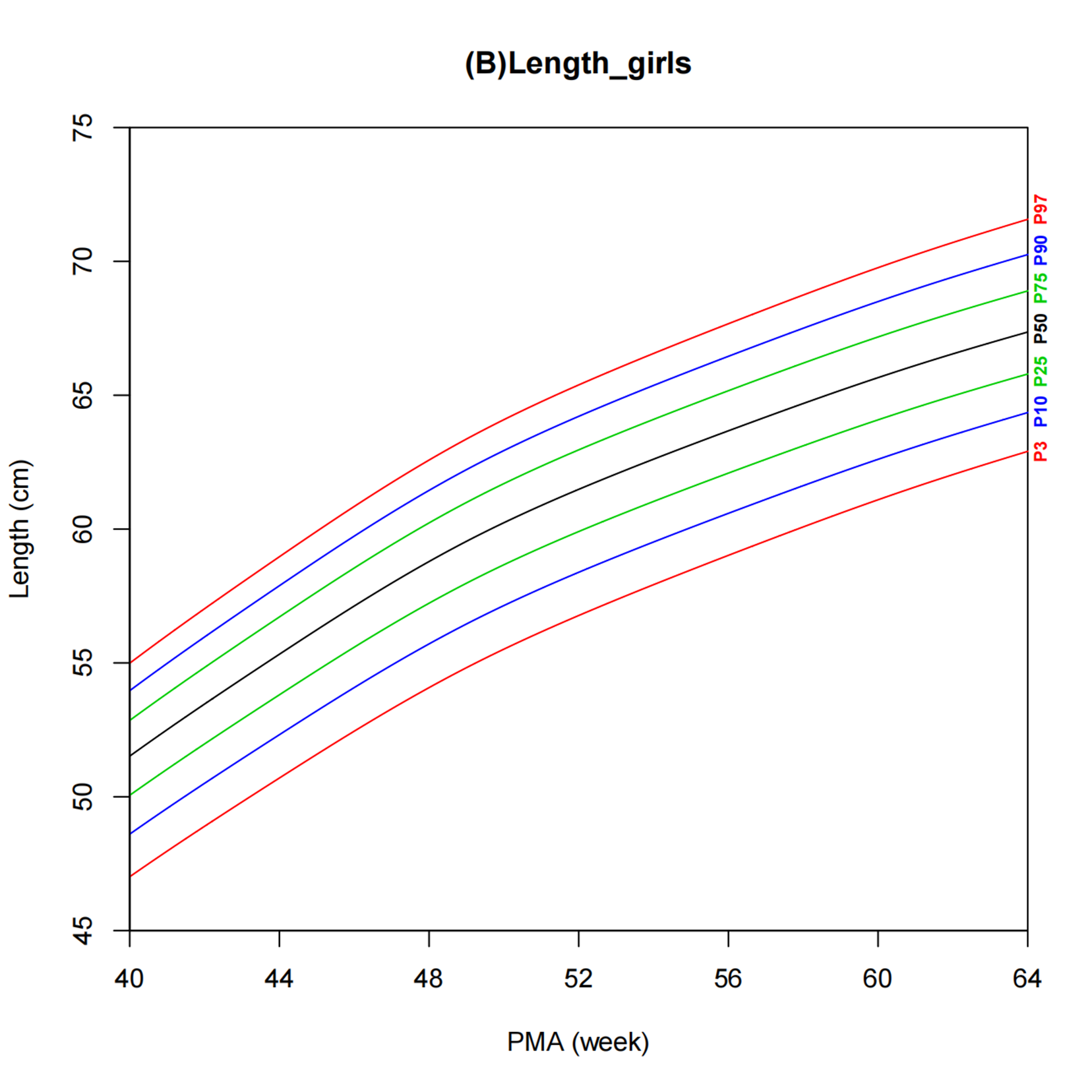

Supplement: Supplementary file 17 [file Image_2.TIF]

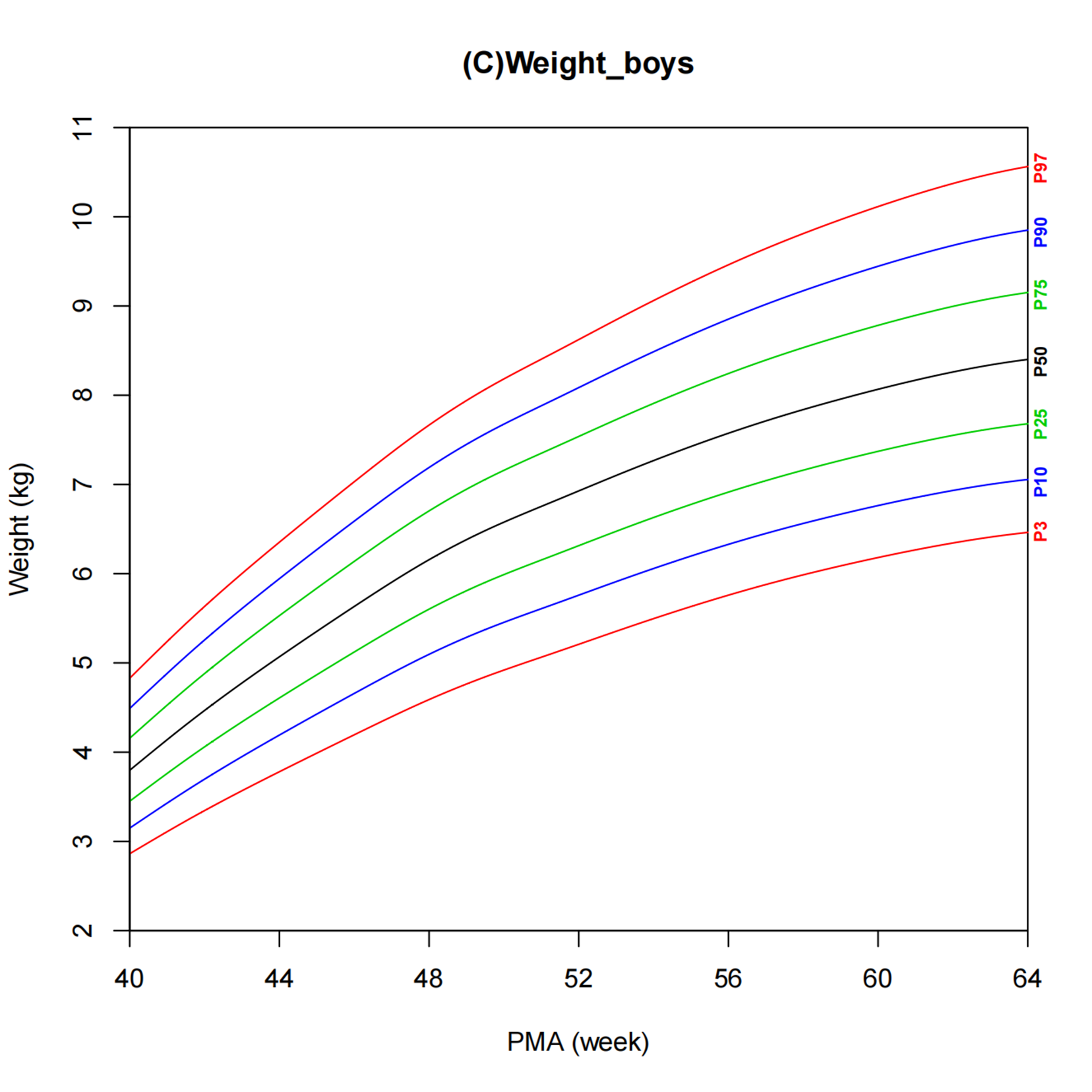

Supplement: Supplementary file 18 [file Image_3.TIF]

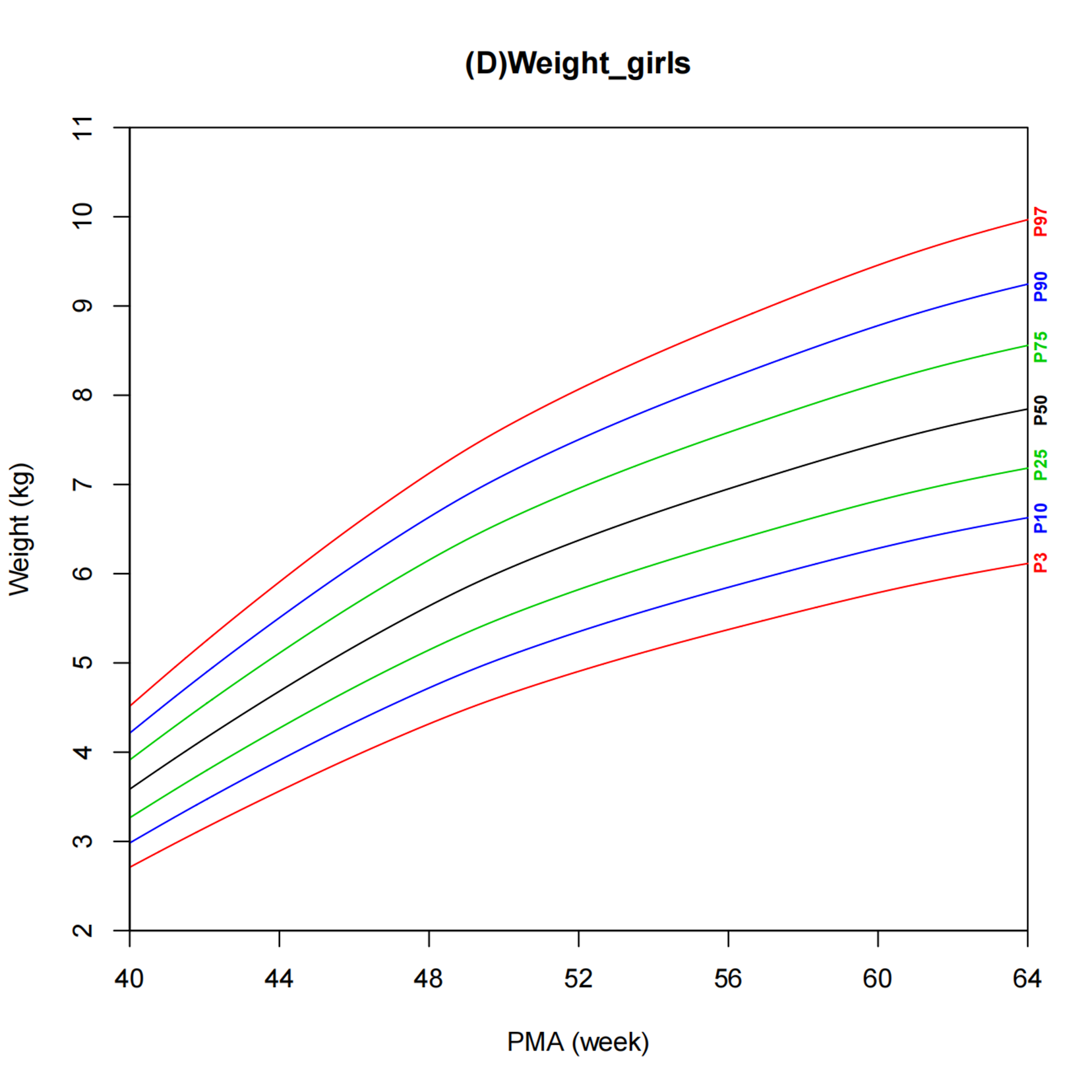

Supplement: Supplementary file 19 [file Image_4.TIF]

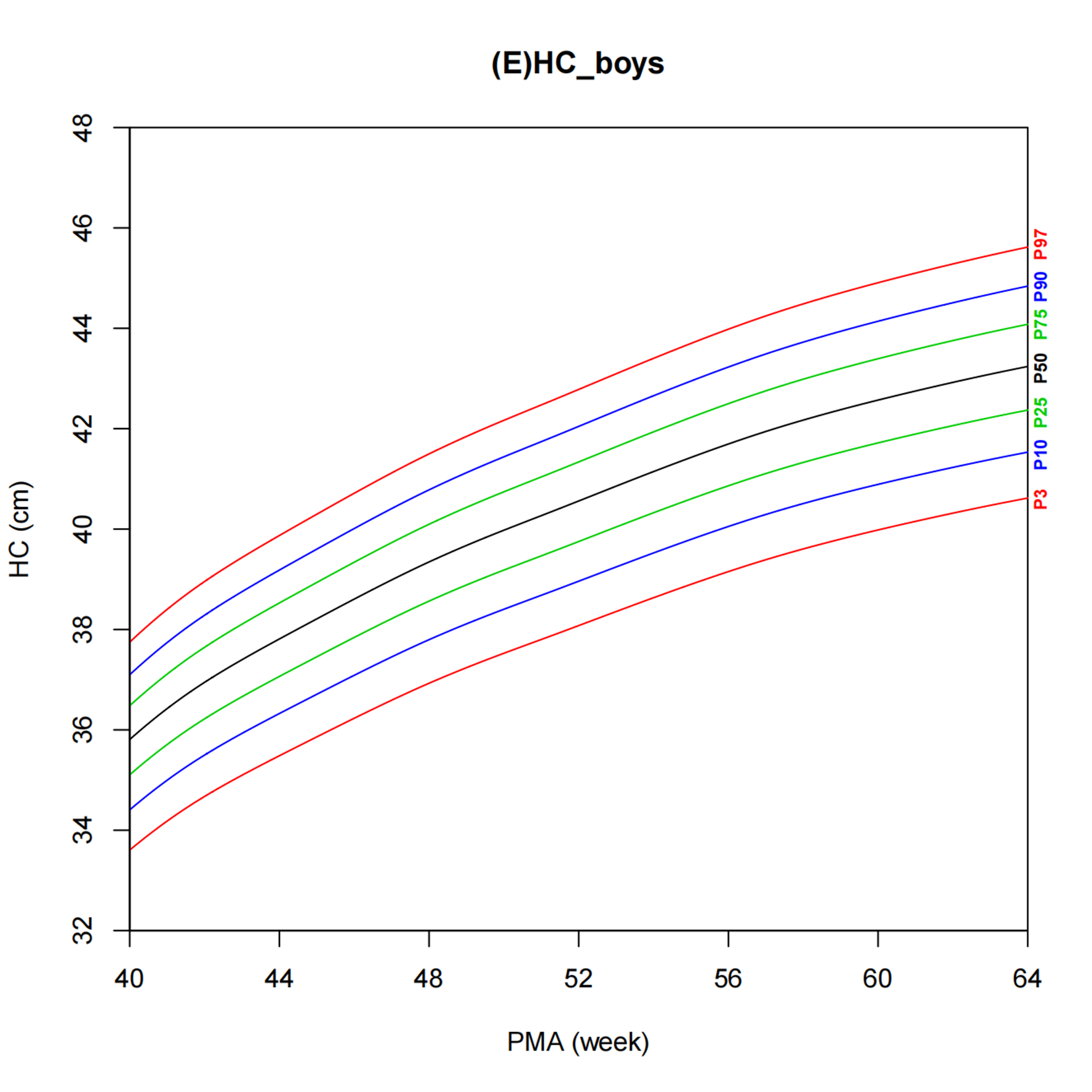

Supplement: Supplementary file 20 [file Image_5.TIF]

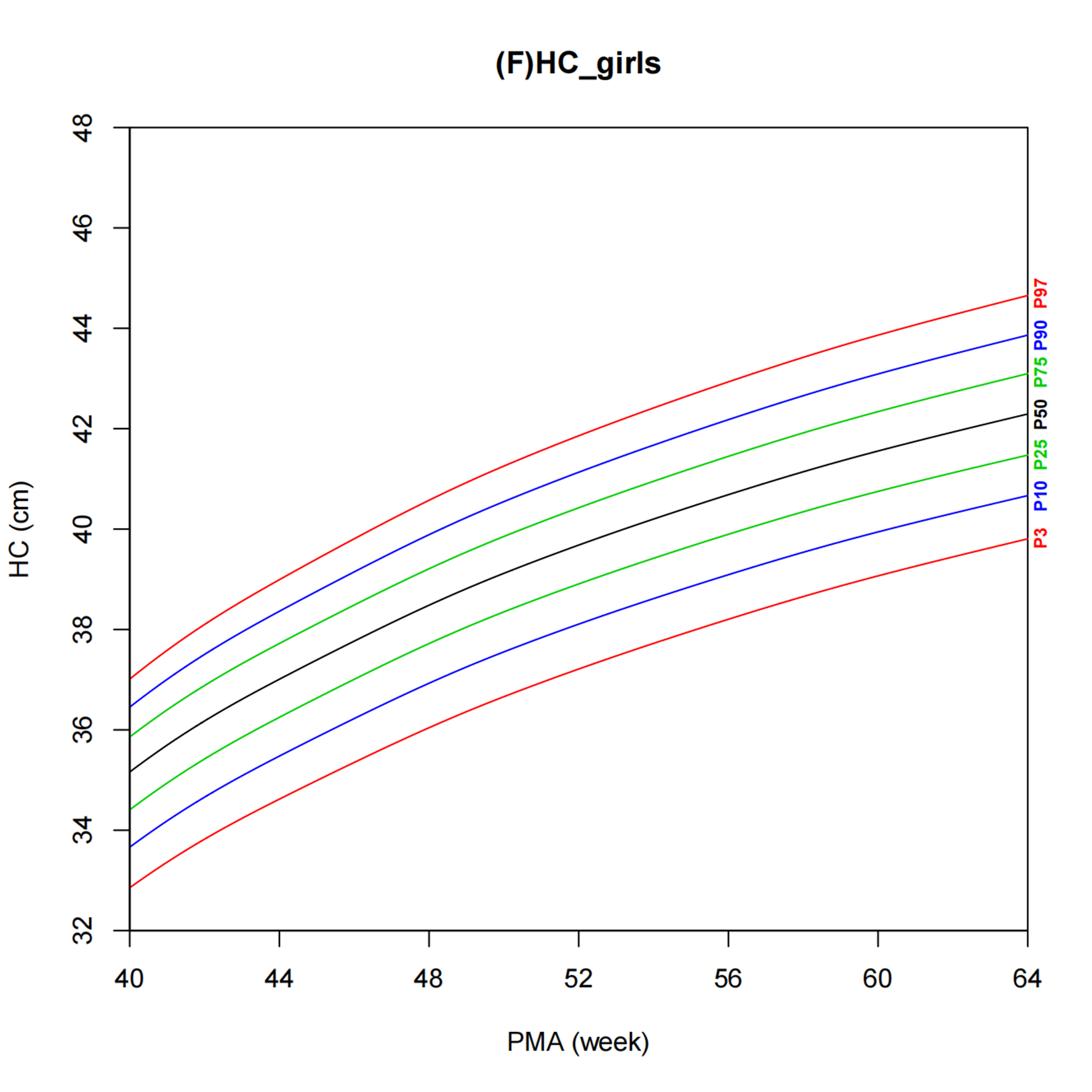

Supplement: Supplementary file 21 [file Image_6.TIF]
